# Left renal cyst – Left duplex kidney with compromised superior renal unit and ectopic ureteral orifice in the prostatic urethra

**Published:** 2013-06-25

**Authors:** D Spinu, O Bratu, V Madan, C Farcas, A Radulescu, R Popescu, D Mischianu

**Affiliations:** “Dr. Carol Davila” Central Military University Emergency Hospital, Bucharest, Urology Ward

**Keywords:** duplex kidney, urethral ectopy, urinary abnormality

## Abstract

The urinary abnormalities are an important health problem. If they are not recognized in due time, they usually lead to the loss of the renal unit function. In many cases, the diagnosis is late and incidental.

Case presentation. We present the case of M.I., a 74-year-old male admitted in our surgical unit with diffuse left lumbar pain, low urinary tract symptoms and slow increase in abdomen volume in the past 4 years. Computer tomography scan and ecography showed a large left lumbar cyst like mass with a dilated supernumerary ureter with ectopic ureteral orifice in the prostatic urethra and apparently normal anatomic inferior renal unit.

The goal was the excision of the “cyst like” mass (superior left renal unit) but because of the anatomical particularities (extensive fibrosis and local topographical changes) total nephrectomy was performed.

Conclusions. Given a normal contralateral kidney, the discovery of a urinary abnormality can be a real challenge, their evolution being a silent one. This type of disease can be suspected only with the development of clinical symptoms.

The anatomic particularities (duplex kidney) together with the long evolution of the disease changed the local topography making the preservation of the inferior left renal unit a difficult, almost impossible task for the surgeon.

## Introduction

The urinary abnormalities are an important health problem, which, if left undiagnosed or untreated, lead to the loss of the respective kidney [1]. Because of the insidious, silent evolution with no specific symptoms, the patient usually addresses to the doctor very late.

 Complete ureteral duplication is an anomaly in which two pelvicalyceal systems drain the same kidney through two separate ureters with two different ureteral orifices. The anomaly occurs when two separate anatomic ureteral buds rotate 180 degrees during their ascension [2,3].

 The ureteral duplication is the most common anomaly of number, the incidence is 0,9%, female to male retio is 1,6 to 1 and is six times more frequent unilaterally than bilaterally.

 This type of anomaly usually coexists with ureteral ectopy [4]. Ectopic ureters are rare, 80% drain the superior ureter of a duplex kidney and the rest (20%) drain single ones. In females, 80% of ectopic ureters coexist with the duplex system, but in males, the great majority associate with the single system. Weigert – Myer rule is of great clinical importance, since he is the one who noticed that the distal orifice drains the upper pole and the cranial orifice drains the lower pole when performing cystoscopy.

The prostatic urethra is the most common localization for the ectopic ureteral orifice (48%) followed by the seminal vesicles (40%), ejaculatory ducts (8%), vas deferens (3%), epididymis (0,5%) and rectum (0,5%) in male patients.

 The vagina (30%), proximal vagina (25%), bladder neck (25%), the uterus and the cervix (5%), the Gartner duct (4%) and the urethral diverticula are the most common localizations in female patients.

 The most important symptom in female patients is the urinary incontinence. Males most often present with urinary tract infections. In addition, they may experience urgency, epididymitis and a lot of non specific symptoms (constipation, abdominal pain, discomfort during ejaculation, infertility).

 Duplex kidney without obstruction has two separate pelvicalyceal units, in most of the cases, patients have no symptoms and often it is an accidental imagistic finding.

 The renal unit drained by the ectopic ureter is in many cases compromised, heminephrectomy or total nephrectomy is the advocated treatment. In the single ectopic ureter, the salvation of the kidney can be tried by other surgical means [5].

 For the pediatric patients, the nephron sparing surgery is necessary. In these cases, heminephrectomy ureteroureterostomy or ureteroneocystostomy is the recommended treatment.

## Case presentation

A 74-year-old patient, M.I., was admitted in our surgical unit with diffuse left lumbar pain, iritative low urinary tract symptoms and slow increase in left abdomen volume (past 4 years). Giant left renal cyst was suspected. 

 Personal history includes operated perforated duodenal ulcer, umbilical hernia, postoperative eventration, diabetes mellitus, extrasystolic ventricular arrhythmia. 

 Biochemistry was normal except for the glucose and chloride blood levels, Computer tomography scan detected “cyst like” mass – compromised superior left renal unit with supernumerary ureter opened in the prostatic urethra.

**Fig. 1:**
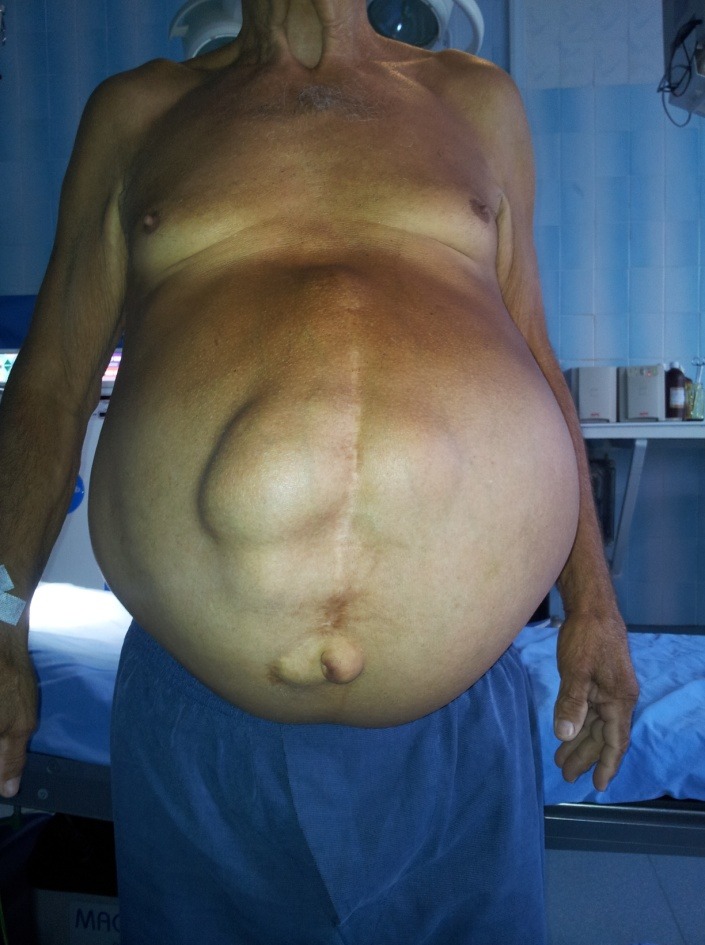
Preoperative aspect (voluminous median eventration, umbilical hernia)

Cystoscopy revealed ectopic left ureteral orifice with prostatic urethral insertion.

**Fig. 2:**
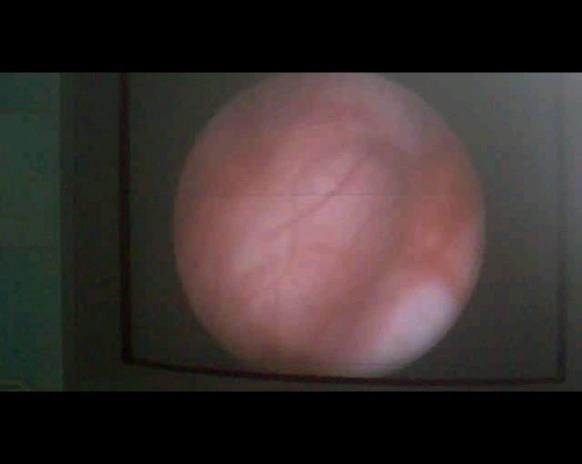
Ectopic left ureteral orifice

**Fig. 3:**
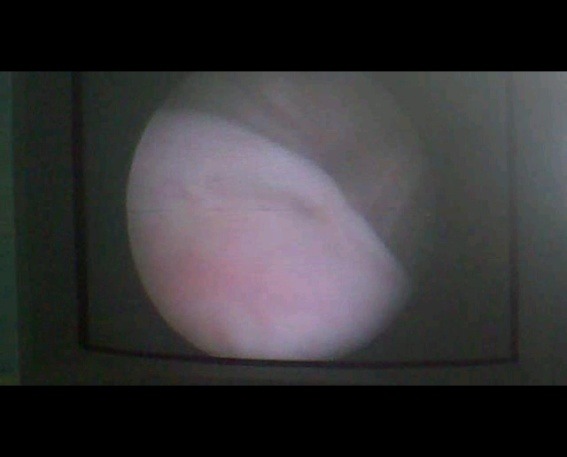
Normal left ureteral orifice

 The patient was diagnosed with left duplex kidney with compromised superior renal unit and ectopic ureteral orifice.

Left nephrectomy by Iterative median incision was performed. Almost 12 liters of clear liquid were punctioned. Excision of the umbilicus with eventration repair was also performed. 

Extensive fibrosis, aberrant vessels, elongated inferior renal artery made the goal of salvaging the inferior renal unit impossible.

**Fig. 4:**
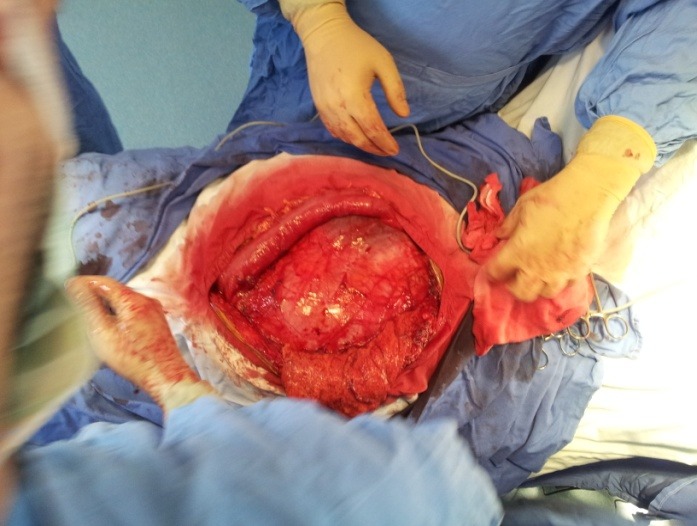
Voluminous “cyst like” mass in the left flank

**Fig. 5:**
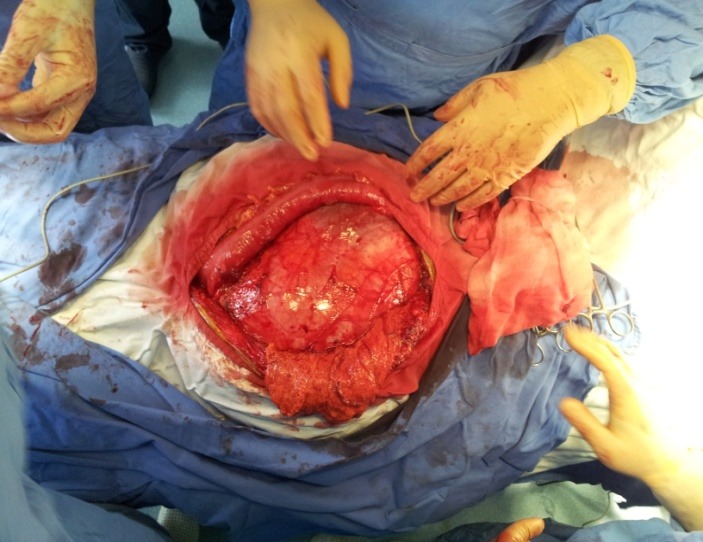
Voluminous “cyst like” mass in the left flank

**Fig. 6:**
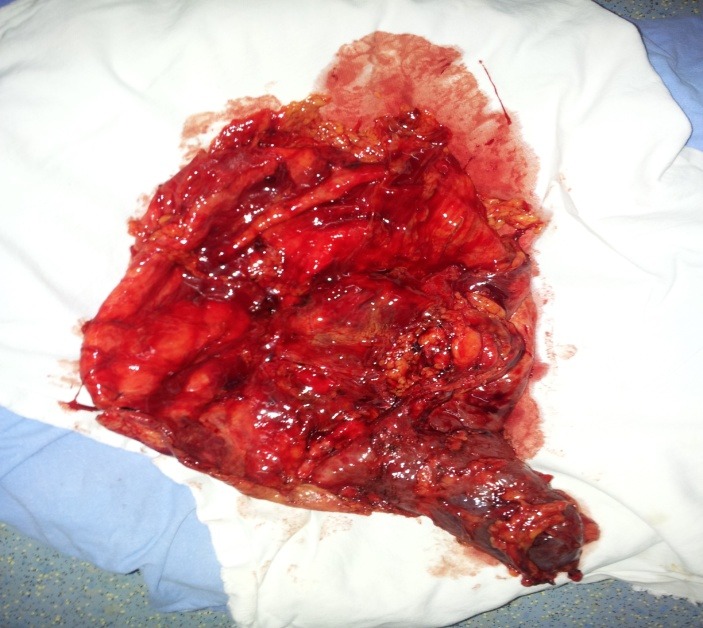
Resected specimen with normal inferior renal unit

**Fig. 7:**
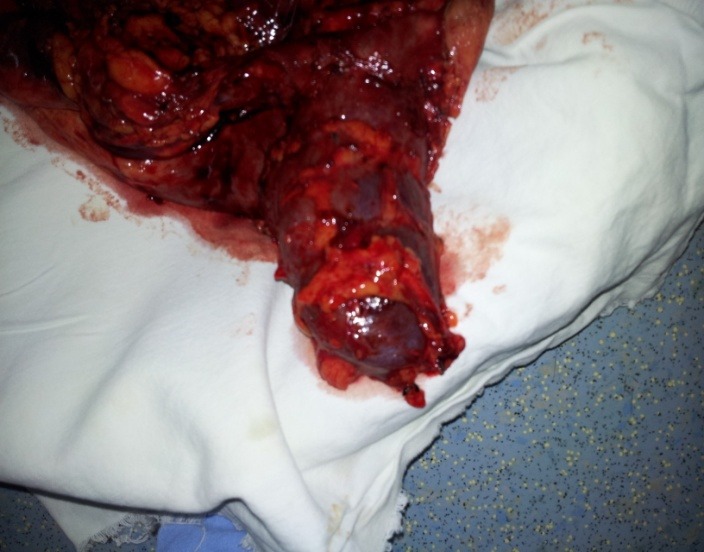
Left inferior renal unit

 Postoperative evolution was simple with intravenous medication, antibiotics and analgesics.

**Fig. 8:**
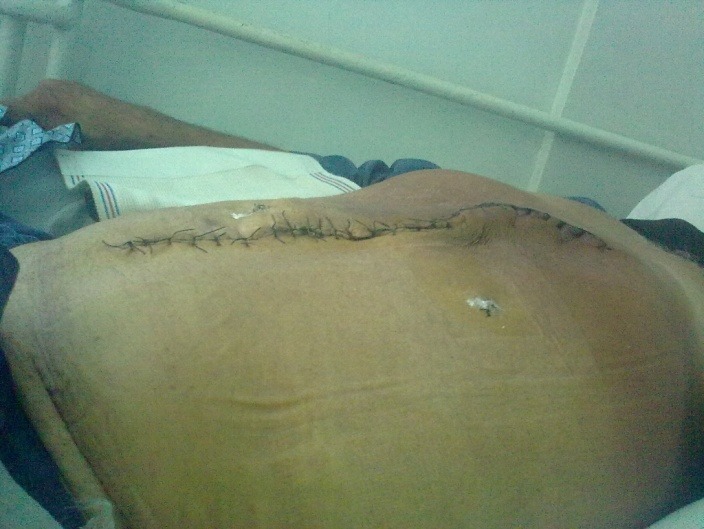
Seven days postoperative aspect (umbilical hernia and eventration repair)

**Fig. 9:**
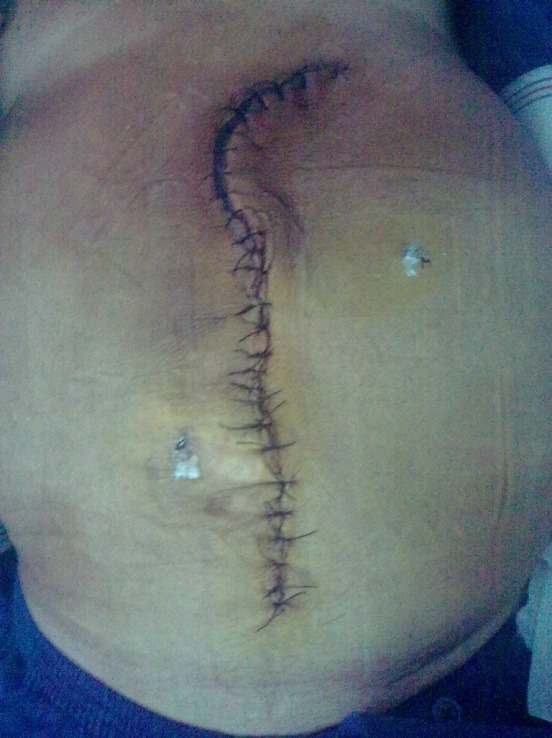
Seven days postoperative aspect (umbilical hernia and eventration repair)

## Discussions

 This type of disease can develop silently with no specific symptoms. The optimal surgical approach is superior nephroureterectomy with inferior renal unit reservation. Subtotal ureterectomy (above the iliac vessels) is an option if no vesicoureteral reflux is noticed. If the vesicoureteral reflux exists, total ureterectomy is recommended. 

 Because of the prolonged evolution, extensive local anatomy alterations were present. Extensive fibrosis and inferior renal pedicle elongation made the objective of salvaging the inferior renal unit an impossible task. 

 We had to evacuate 12 liters of sterile clear liquid before extracting the specimen. 

 Histopathologic examinations revealed a renal tissue, non-specific chronic inflammation, necrosis areas associated with intense fibrosis. 

 We presented this case because the association of the two urinary abnormalities, duplex kidney with ectopic ureteral orifice in the prostatic urethra is rare and often undertreated. 

**Acknowledgement**

 This paper is supported by the Sectoral Operational Programme Human Resources Development (SOP HRD) 2007-2013, financed from the European Social Fund and by the Romanian Government under the contract number POSDRU/107/1.5/S/82839",
